# A multilevel impact study of Chinese adolescents’ sports participation based on ecological models of health behavior: a structural equation model analysis

**DOI:** 10.3389/fpsyg.2025.1593491

**Published:** 2025-10-03

**Authors:** Sha Ge, Yifan Zhao, Haoze Song, Xuepeng Guo, Chao Song

**Affiliations:** ^1^College of Sports Science, Tianjin Normal University, Tianjin, China; ^2^School of Political Science and Administration, Tianjin Normal University, Tianjin, China; ^3^Department of Physical Education, Tiangong University, Tianjin, China

**Keywords:** adolescent, ecological models of health behavior, structural equation modeling, sports participation, Chinese adolescent

## Abstract

Despite the growing awareness of the health benefits of physical activity participation among adolescents, physical inactivity remains a pressing concern for many adolescents around the globe. Using Ecological models of health behavior as a guide, this study investigated the combined effects of environmental, organizational, interpersonal, and individual factors on Chinese junior high school students’ sport participation. A four-level structural equation model (SEM) integrating environmental, organizational, interpersonal, and individual factors was developed for 780 students (51.79% boys, 48.21% girls) from nine middle schools in Heping District of Tianjin. The model showed acceptable fit (CMIN/DF = 2.601, GFI = 0.949, CFI = 0.971, TLI = 0.965, RMSEA = 0.045), and correlation analyzes indicated that sport participation was moderately positively correlated with personal, interpersonal, organizational, and environmental factors (*r* = 0.476–0.531, *p* < 0.01). Structural equation modeling further confirmed that Environmental Factors (Env.F), Organizational Factors (Org.F), Interpersonal Factors (Int.F) and Individual Factors (Ind.F) had significant direct and indirect effects on sport participation (SP). Multiple and interlocking mediation paths emerged, indicating partial mediation between the four levels. “Int.F → Ind.F → SP” showed a strong indirect effect (β = 0.124, 95% CI [0.043, 0.221]), emphasizing the critical role of interpersonal support and personal confidence in shaping youth sport participation. Further, the most extensive chain, “Env.F → Org.F → Int.F → Ind.F → SP,” also emerged as a valid path, with an indirect effect of β = 0.027 (95% CI [0.010, 0.052]). The results suggest the need for a multilevel intervention that coordinates environmental and organizational resources, strengthens family and peer support, and fosters individual self-efficacy to ultimately promote sustained youth participation in sports.

## 1 Introduction

The issue of global concern regarding the decline in the physical health and well-being of adolescents has garnered significant attention from research communities. The prevalence of obesity and overweight among adolescents, often attributed to insufficient physical activity, unhealthy eating patterns, and adverse lifestyle habits, underscores the critical importance of addressing this issue. Research conducted by Dong et al. (2023) on the 2019 National Student Physical Activity and Health Survey revealed that over 80% of adolescent individuals fail to achieve the recommended daily exercise guideline of at least one hour of physical activity (Dong et al., 2023). Furthermore, a study revealed that two out of three adolescents were diagnosed with obesity or overweight (Kumari Ekanayake et al., 2023). Insufficient physical activity in adolescents has been associated with an increased risk of developing non-communicable diseases, such as cardiovascular disease, diabetes, or cancer (World Health Organization [WHO], 2024). Additionally, a survey conducted by the World Health Organization (WHO) in 2024 indicated that insufficient physical activity has emerged as a major risk factor for global mortality (World Health Organization [WHO], 2024). The rise in academic pressure and the pervasive use of electronic devices have contributed to this issue. Adolescents are increasingly spending more time on electronic devices, and their daily activities are gradually shifting from physical activity to sedentary entertainment. This shift is likely to have a negative impact on their physical and mental health.

Physical activity is a non-pharmacological intervention that can prevent and improve physical health, as well as psychological well-being (Wang et al., 2024). Al Zaki et al. (2023) and Calcaterra et al. (2022) have demonstrated that regular participation in sporting activities has a positive impact on cardiovascular health and metabolic function. In addition, these activities play a role in the prevention and delay of health complications associated with adolescent obesity. Furthermore, these activities have been found to improve the emotional well-being of adolescents, reducing symptoms of anxiety and depression (Recchia et al., 2023). However, many adolescents still do not engage in sports regularly, and this phenomenon requires further investigation to understand its underlying causes.

### 1.1 Multilevel influences on adolescent sports participation

Adolescence is a critical transitional period between childhood and adulthood, a phase during which both physical and psychological development undergo rapid changes. Research has demonstrated that regular participation in physical activities can significantly enhance not only the physical health of adolescents, but also their psychological well-being, social competence, and overall quality of life (Biddle et al., 2019; Vaquero-Solís et al., 2021; Villafaina et al., 2021). It is imperative to acknowledge that the participation of adolescents in physical activities is not solely influenced by Individual Factors (Ind.F), but rather is shaped by a multitude of environmental influences. Physical activity is not merely a form of exercise, but rather a lifestyle choice, influenced by external factors such as societal culture, sports policies, and available sports resources (Kerstetter and Kovich, 1997). It is imperative to acknowledge that participation in sports among adolescents is not solely influenced by Ind.F but is also shaped by a multitude of environmental influences. Sports participation transcends its mere physical activity nature and manifests as a lifestyle choice, with its forms and substance influenced by external environmental factors, including societal cultural influences, sports policies, and the availability of sports resources (Kerstetter and Kovich, 1997). This influence manifests in various aspects of sports participation, such as the frequency, quality, and type of sports engaged in, including competitive sports, adherence to sports rules, and sports cultural interactions. Consequently, when studying sports participation among adolescents, it is insufficient to focus solely on individual behaviors or single environmental factors. Instead, a more comprehensive and multifaceted interactive framework should be employed to explore these phenomena.

### 1.2 Ecological models of health behavior

This study is grounded in the ecological models of health behavior (Sallis et al., 2015), which posits that behavior is shaped by multiple interacting levels: individual, interpersonal, organizational, environmental, and policy. In line with our measurement scope and modeling objectives, we focus on the first four levels; the policy layer is treated as a distal contextual factor that influences the others, given that our sample comprises nine schools within a single administrative district operating under largely uniform national, provincial, and municipal policies. Within this framework, we employ structural equation modeling (SEM) to examine the direct and indirect (chain) effects of the four levels on adolescents’ sports participation and to compare the relative strengths of the pathways.

### 1.3 Ecological determinants and mediating mechanisms in adolescent sports participation

Prior studies (Ge, 2012; Hu et al., 2021; van Sluijs et al., 2021) examined four categories of factors: physiological, psychological, sociocultural, and environmental. These studies investigated the influence of these factors on adolescents’ physical activity levels through ecological models of health behavior. However, these studies focused mainly on descriptive analyzes of the influencing factors and failed to elucidate their complex interrelationships and mechanisms of action. The national surveys also believe that rapidly changing digital media exposure and academic pressures further complicate adolescents’ participation in regular exercise (Chen et al., 2020). This suggests a lack of understanding of the precise mechanisms by which family environment, peer support, and organizational structure interact to influence adolescents’ activity behaviors. This gap indicates a need for a more comprehensive and mechanism-oriented approach to studying adolescent physical activity, especially in the Chinese context.

This study addresses a critical gap in the Chinese context by examining how individual, interpersonal, organizational, and environmental factors jointly influence adolescents’ sports participation. Using structural equation modeling (SEM), we systematically evaluate the ecological models of health behavior and estimate direct and indirect effects across individual, interpersonal, organizational, and environmental levels. Individual confidence and expectations are considered as indicators of psychological intention, while family support, school curriculum, and community environments represent external supports and constraints. By integrating these dimensions within a unified framework, the study provides new theoretical and empirical insights into the mechanisms that encourage adolescents’ willingness and sustained engagement in physical activity. The findings not only advance understanding of sports participation in China but also offer evidence-based guidance.

### 1.4 Paths and hypotheses

The ecosystem theory posits that individual behavior is influenced by multiple levels of environmental factors, including individual, physiological, psychological, sociocultural, and environmental aspects. These factors interact with each other to create complex ecological models of health behavior that provides a deeper understanding of the driving mechanisms of adolescent sports participation. Adolescents are in a stage of rapid physical and mental development, and sports participation is important for their comprehensive development. In light of these considerations, the present study has structured its analysis around the following four-level categorization of factors influencing adolescent sports participation (see Table 1).

**TABLE 1:** Related factors.

Ind. F	Physiological Factors (Phy. F): Age; Gender; Grade.
	Psychological Factors (Psy. F): Self-assessment of Confidence in Sports Participation (SAC); Positive and Negative Outcome Expectations in Sports Participation (PNOE).
Int. F	Parents: Parental Support for Sports Participation (PS);
	Friends (Classmates): Friends’ Support for Sports Participation (FS).
Org. F	School Physical Education Organization (PE);
	the Influence of School on Sports Participation (IS).
Env. F	Family Environment Influence (FEI): Ownership and Use of Sports Equipment in the Family (OUSEF);
	Community Public Environment (CPE): Public Service Facilities Around the Community (PSAC);
	Community Residential Environment (CRE): Assessment of the Attractiveness of the Residential Environment (AARE);
	Community Sports Service Environment (CSSE): Sports Service Facilities Around the Community (SSFAC).

Env.F: Environmental Factors; Int.F: Interpersonal Factors; Ind.F: Individual Factors; Org.F: Organizational Factors; SP: Sports Participation.

#### 1.4.1 Individual factors

At the individual level, factors such as physical attributes (age, gender, grade level) and psychological elements (self-confidence, positive and negative outcome expectations) are considered. Physical development and social role changes can influence enthusiasm for sports. Gender differences may lead to preferences for different sports and frequency of participation. Increasing academic pressure with grade level may reduce available time and sports options, affecting overall participation (Hulteen et al., 2017; Tammelin et al., 2003; Frömel et al., 2020). Psychologically, self-efficacy and positive outcome expectations often predict engagement, while anticipated negative outcomes may reduce motivation (McGrane et al., 2017; Crozier and Spink, 2017; Gyurcsik et al., 2015). Hypothesis: H1 Ind.F have a direct impact on Sports participation.

#### 1.4.2 Interpersonal factors

Interpersonal factors emphasize family and peer support. Parents’ encouragement and resources can enhance motivation, and friends’ recognition and companionship also influence participation (Craggs et al., 2011; Fitzgerald et al., 2012; Sawka et al., 2013; Olivares et al., 2015). Interpersonal factors can directly affect participation and indirectly influence individual psychological variables such as self-efficacy and outcome expectations (Corsano et al., 2006; Hamilton et al., 2017). Hypotheses: H2a Interpersonal factors have a direct effect on Sports participation; H2b Interpersonal factors have a direct effect on Ind.F; H5 Interpersonal factors influence Ind.F, which in turn lead to sports participation.

#### 1.4.3 Organizational factors

At the organizational level, school physical education and overall school environment influence sports participation through curricula, extracurricular activities, and social practices (Nahas et al., 2003; Trudeau and Shephard, 2008; Diamant et al., 2011; Mitchell et al., 2015). Organizational factors can directly impact participation, interpersonal factors, and individual factors (Drolet et al., 2013; Feraco et al., 2023; Singla et al., 2021). They may also indirectly influence participation through multiple mediation pathways involving interpersonal and individual factors. Hypotheses: H3a Organizational factors have a direct impact on Sports participation; H3b Organizational factors have a direct impact on Interpersonal factors; H3c Organizational factors have a direct impact on Ind.F; H6 Organizational factors influence Ind.F, which subsequently lead to sports participation; H8, H10 Organizational factors influence interpersonal factors, which in turn shape Ind.F, ultimately leading to sports participation.

#### 1.4.4 Environmental factors

**FIGURE 1:**
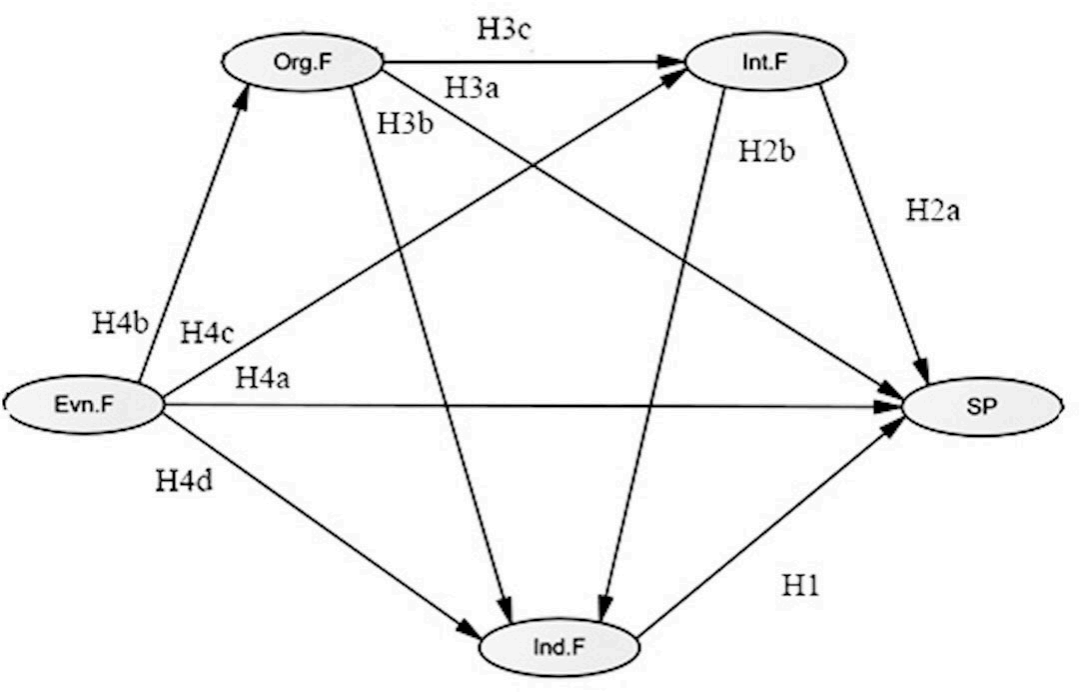
Hypothesized model. Env.F: Environmental Factors; Ind.F: Individual Factors; Int.F: Interpersonal Factors; Org.F: Organizational Factors; SP: Sports Participation.

Environmental factors include family, community, and public sports resources. Availability and use of sports equipment, public facility quality, living environment comfort, and diversity of community sports services directly affect sports participation (Hu et al., 2021; Ding et al., 2011; Durant et al., 2009; Rodríguez et al., 2012). Environmental factors also shape organizational and interpersonal contexts, influencing social networks, psychological perception, and self-efficacy (Dai et al., 2022; Sun et al., 2022; Odufuwa et al., 2019). Hypotheses: H4a Environmental factors have a direct impact on Sports participation; H4b Environmental factors have a direct impact on Organizational factors; H4c Environmental factors have a direct impact on Interpersonal factors; H4d Environmental factors have a direct impact on Ind.F; H7, H9, H11, H12, H13, H14, H15 specify indirect paths through organizational and interpersonal mediation. The multi-level model and hypotheses are illustrated in Figure 1 (see Figure 1, Table 2).

**TABLE 2:** Hypothetical content.

Number	Hypothetical content
**Direct effects**	
H1	Ind.F have a direct positive effect on SP
H2	Int.F have a direct positive effect on SP
	Int. F positively influence Ind. F
H3	Org. F have a direct positive effect on SP
	Org. F positively influence Int. F
	Org. F positively influence Ind. F
H4	Env. F have a direct positive effect on Sports Participation SP
	Env. F positively influence Org. F
	Env. F positively influence Int. F
	Env. F positively influence Ind. F
**Indirect effects**	
H5	Int. F influence Ind. F, which in turn affect SP
H6	Org. F influence Ind. F, which in turn affect SP
H7	Env. F influence Ind. F, which in turn affect SP
H8	Org. F influence Int. F, which then influence Ind. F, ultimately affecting SP
H9	Env. F influence Int. F, which then influence Ind. F, ultimately affecting SP
H10	Org. F influence Int. F, which in turn affect SP
H11	Env. F influence Int. F, which in turn affect SP
H12	Env. F influence Org. F, which then influence Int. F, ultimately affecting SP
H13	Env. F influence Org. F, which in turn affect SP
H14	Env. F influence Org. F, which then influence Ind. F, ultimately affecting SP
H15	Env. F influence Org. F, which influence Int. F, subsequently influencing Ind. F, and ultimately affecting SP

Env. F: Environmental Factors; Int. F: Interpersonal Factors; Ind. F: Individual Factors; Org. F: Organizational Factors; SP: Sports Participation.

## 2 Materials and methods

### 2.1 Participants

Heping District is one of the six most representative inner-city districts in Tianjin, China, located in the central area of the city. A stratified random sampling method was used to recruit junior high school students. Nine schools were randomly selected from all junior high schools in Heping District, covering the first, second, and third sub-districts. From the initial survey, 803 questionnaires were distributed. Ultimately, 780 valid responses were obtained, achieving a response validity rate of 97.1%.

All participants and their parents or legal guardians provided written informed consent prior to the study. The research was conducted in accordance with the Declaration of Helsinki and approved by the Ethics Committee of Tianjin Normal University, China (Approval No. 2023102301, October 23, 2023). Consent forms signed by parents or guardians confirmed permission for child participants to take part in the study, and all collected information was treated with strict confidentiality.

### 2.2 Outcome measures

All outcomes in this study are considered primary. The design is exploratory regarding underlying mechanisms, aiming to understand how these outcomes might respond to interventions and to reveal possible influences for future theoretical and practical guidance. The translation and cultural adaptation followed standard cross-cultural procedures (Beaton et al., 2000; Brislin, 1970).

#### 2.2.1 Sports participation

“Healthy People 2020 goals” set targets for improving adolescents’ physical activity levels (U S. Department of Health and Human Services, 2000). Drawing on and modifying three items from that report, we captured the physical activity patterns of adolescents over the past 7 days: 1. “On how many of the past 7 days did you engage in at least 60 min of physical activity?” 2. “On how many of the past 7 days did you participate in more than 30 min of exercise that made you sweat or breathe heavily?” 3. “On how many of the past 7 days did you engage in at least 30 min of daily activities that did not make you sweat or breathe heavily?” Each question was scored on an 8-point Likert scale from 1 (0 days) to 8 (7 days). To ensure cultural appropriateness for Chinese adolescents, the items were translated into Chinese via forward translation, reviewed by a three-member expert panel (public health, PE pedagogy, measurement), back-translated by an independent bilingual researcher, and refined through cognitive interviews with a small sample of students. Pilot testing confirmed comprehension and adequate response variability, with Cronbach’s α = 0.766, indicating acceptable internal consistency.

#### 2.2.2 Individual factors

The individual-level variables influencing physiological factors included date of birth (calculated age), gender, grade level, net height, and net weight. These data were obtained through self-report and on-site measurements by trained researchers. The primary objective was to assess basic individual physical fitness and physiological characteristics. A substantial body of research has demonstrated that factors such as age, gender, and physical characteristics exert a considerable influence on adolescents’ engagement in sports (Sallis et al., 2000; Dumith et al., 2011). Psychological influences. Two questionnaires were developed for the present study: one for the self-assessment of confidence in physical activity participation, comprising 17 items (e.g., “I can participate in physical activity no matter how tired I am”). The second questionnaire was designed to assess expectations of positive and negative outcomes associated with physical activity participation, comprising 16 items (e.g., “physical activity will make me fitter”). Scales are based on Bandura’s framework (Bandura, 1997) and prior adolescent PA studies (Motl et al., 2007; Dishman et al., 2009). Items were drafted in Chinese, reviewed by an expert panel, refined via cognitive interviews with students, and pilot-tested to ensure clarity, cultural relevance, and age appropriateness. Each question was scored on a 5-point Likert scale ranging from 1 (strongly disagree) to 5 (strongly agree). Items were drafted in Chinese, reviewed by an expert panel, refined via cognitive interviews with students, and pilot-tested to ensure clarity, cultural relevance, and age appropriateness.

#### 2.2.3 Interpersonal factors

The interpersonal level was assessed by two questionnaires: Parental Support and Friend Support. First, the children were asked to rate 20 items based on the level of parental support for sport participation on a 5-point Likert scale, including the item “My family does sport with me.” Subsequently, a 13-item questionnaire was developed to assess the level of support from friends (or classmates) for sports participation, also rated on a 5-point Likert scale ranging from 1 (never) to 5 (a lot). Examples of items include “Friends (or classmates) participate in sports with me.” Items were adapted from established constructs in adolescent PA research (Sallis et al., 2000; Duncan et al., 2005; Pugliese and Tinsley, 2007). Chinese-language versions underwent expert review, student cognitive interviews, and pilot testing to ensure comprehension, cultural appropriateness, and age suitability, capturing perceived social support effectively in the Chinese context.

#### 2.2.4 Organizational factors

The impact of the organization of physical education instruction in schools on sport participation was measured by participants filling out a questionnaire consisting of 9 items, such as the impact of the physical education teacher’s teaching philosophy. Participants were asked to rate their perceived level of influence on a 5-point Likert scale ranging from 1 (never) to 5 (a lot). Items were informed by prior school-based PA research (Fox and Harris, 2003; Dobbins et al., 2013; Yi et al., 2016) and adapted for Chinese junior-high contexts. The cultural adaptation process included expert review and pilot testing to ensure relevance and clarity for local school routines.

#### 2.2.5 Environmental factors

The present study established four subcategories at the environmental level to measure the influence of four aspects: home environment, community public environment, community residential environment, and community sports service environment. The Ownership and Use of Sports Equipment in the Family (OUSEF), a seven-item instrument, solicited information regarding the ownership and utilization of sports equipment within households, with subjects rating the frequency of this practice on a five-point Likert scale ranging from 1 (none) to 5 (more than once a week). The Public Service Facilities Around the Community (PSAC), a four-item instrument, characterized the state of public service facilities in the vicinity. The community public environment was evaluated on a 5-point Likert scale (1 = strongly disagree, 5 = strongly agree). The 4-item community public environment questionnaire described the public service facilities around the community, such as “It is very close to the shops in the neighborhood from your house.” It was evaluated on a 5-point Likert scale (1 = strongly disagree, 5 = strongly agree). Likert scale (1 = strongly disagree, 5 = strongly agree); and the 4-item community living environment questionnaire, which described the aesthetics of the living environment, such as (There are trees planted on both sides of the road in your community) was evaluated on a 5-point Likert scale (1 = strongly disagree, 5 = strongly agree). Finally, a 4-item questionnaire was used to describe the impact of environmental facilities for community sports services, such as the distribution of fitness equipment in residence affecting sports participation. In this case, subjects were asked to rate their self-perceptions on a Likert scale ranging from 1 (strongly disagree) to 5 (strongly agree). Items were based on ecological models of health behavior (Sallis et al., 2015) and empirical PA studies (Ding et al., 2011; Timperio et al., 2006), drafted in Chinese, reviewed by experts, cognitively interviewed with students, and pilot-tested to ensure clarity and cultural relevance.

#### 2.2.6 Reliability and validity procedures

We assessed internal consistency using Cronbach’s α (acceptable ≥ 0.70), sampling adequacy with KMO and Bartlett’s test, and evaluated the measurement model via CFA (reporting CFI/TLI/IFI, RMSEA) and convergent/discriminant validity (CR ≥ 0.70, AVE ≥ 0.50; Fornell–Larcker).

### 2.3 Procedure

This study combined online surveys with offline organization, with the research team collaborating with local schools to distribute questionnaires to students. The questionnaire included an informed consent form, a QR code for the online questionnaire, and contact information. Schools distributed the questionnaire link or QR code to students, who completed the questionnaire after reading and signing the informed consent form. The follow-up data was collected and organized by the research team.

### 2.4 Data analysis

In this study, descriptive statistics were initially conducted on the collected valid questionnaires to calculate the mean, standard deviation, skewness, and kurtosis of each main variable (sports participation, individual factors, interpersonal factors, organizational factors, environmental factors) to obtain a preliminary picture of the data distribution characteristics (Field, 2024). The scale was then subjected to a reliability analysis using Cronbach’s α coefficient, a measure of internal consistency. According to Tavakol and Dennick (2011), α ≥ 0.9 indicates excellent internal consistency, 0.8–0.9 is good, 0.7–0.8 is acceptable, 0.6–0.7 indicates the need for further revision, and below 0.6 indicates poor internal consistency of the scale. To assess the validity of the scale, SPSS 26.0 was used to perform KMO and Bartlett’s test of sphericity to evaluate the suitability of the data. A KMO of ≥0.9 indicates that the scale is well suited for factor analysis, 0 A KMO of 0.9 or higher is considered adequate for factor analysis, while values between 0.7 and 0.9 are deemed suitable for this purpose. However, if the KMO falls below 0.5, the scale should be disregarded. For dichotomous variables such as gender, if the Bartlett’s test is significant at the *p* < 0.05 level, it indicates that there is a high enough correlation between the items for further factor analysis (Hair et al., 2014). For dichotomous variables such as gender, independent samples t-tests were utilized to examine differences across gender populations if each of the main variables met normal distribution, and for grade level (a multicategorical variable), a one-way analysis of variance (ANOVA) was used to assess differences from each of the main variables (Park, 2009). Following the completion of reliability and validity tests, the present study underwent a confirmative factor analysis (CFA) via AMOS 24.0, with primary reference to the chi-square/degrees of freedom ratio (Chi-square to degrees of freedom, CMIN/DF), the Goodness-of-Fit Index (GFI), the Value-Added Fit Index (Incremental Fit Index (IFI), and the Tucker-Lewis Index. (TLI), Comparative Fit Index (CFI), and Root Mean Square Error of Approximation (RMSEA). Typically, a CMIN/DF ratio less than 5, along with a GFI/IFI/TLI/CFI greater than 0.8 or 0.9, is considered acceptable or good. Additionally, an RMSEA less than 0.08 also indicates a satisfactory model fit (West et al., 2012). Convergent validity and discriminant validity are further examined if the fit indicators are deemed acceptable. A combined reliability (CR) higher than 0.70 usually indicates that the items are in good agreement with the latent variables, and an Average Variance Extracted (AVE) higher than 0.50 indicates good convergent validity. If multiple paths are present, discriminant validity is determined by comparing the square root of the AVE of each factor to the correlation coefficients between the factors themselves. If the square root exceeds the correlation coefficient, the AVE of the factor is compared with the correlation coefficient of the other factors. If the square root exceeds its correlation coefficient, it indicates effective differentiation between the factors (Shrestha, 2021). Pearson’s correlation analysis was then employed to explore the interrelationships between the primary variables. A *p*-value of less than 0.05 and a correlation coefficient greater than 0 indicate a positive correlation, while a value less than 0 indicates a negative correlation (Sedgwick, 2012). Finally, this study constructed a SEM and used AMOS 24.0 to test the model’s fit. The indicators of CMIN/DF, GFI, IFI, TLI, CFI, and RMSEA were met if they met the acceptable standards. Stage of path analysis, exploring the role of the relationship between the latent variables through the level of significance (*p*-value) and standardized path coefficients; and concurrently, we used the Bootstrap sampling method (5,000) to analyze the relationship between the latent variables and the path coefficients. Mediation effects were assessed within the structural equation model using AMOS 24.0 with 5,000 bootstrap samples. A non-zero confidence interval for the indirect effect indicates the presence of mediation, which was combined with the direct effect to determine whether the mediation was full or partial (Preacher and Hayes, 2008).

## 3.Results

### 3.1 Participation

The sample consisted of 780 middle school students (51.79% male; 48.21% female) distributed across three grades: 7th grade (34.36%), 8th grade (34.87%), and 9th grade (30.77%). Anthropometric measurements showed normal distribution (height: Skewness = 0.354, Kurtosis = 1.254; weight: Skewness = 1.207, Kurtosis = 3.165), with mean height of 167.6 cm (SD = 8.37) and mean weight of 56.15 kg (SD = 11.31). All study variables demonstrated acceptable normality (Skewness range: 0.112–0.827; Kurtosis range: 0.157–0.839), with mean scores as follows: sports participation (*M* = 4.876), Ind.F (*M* = 3.851), interpersonal factors (*M* = 3.645), organizational factors (*M* = 3.645), and environmental factors (*M* = 3.720) (see Table 3).

**TABLE 3:** Participants’ characteristics.

Items	Categories		N	Percent (%)							
**Frequency**											
Gender	Male		404	51.79							
	Female		376	48.21							
Grade	Junior high school grade one		268	34.36							
	Junior high school grade two		272	34.87							
	Junior high school grade three		240	30.77							
Total			780	100							
**Descriptive statistics**											
	***N***	**Minimum**	**Maximum**	**Mean**	**Std. Deviation**		**Skewness**			**Kurtosis**	
	**Statistic**	**Statistic**	**Statistic**	**Statistic**	**Statistic**		**Statistic**	**Std. Error**		**Statistic**	**Std. Error**
Net height	780	140	216	167.6	8.37		0.354	0.088		1.254	0.175
Net weight	780	76	260	112.3	22.62		1.207	0.088		3.165	0.175
SP	780	1	8	4.876	1.709		−0.112	0.088		cc	0.175
Ind.F	780	1.121	5	3.851	0.824		−0.827	0.088		0.187	0.175
Int.F	780	1.455	5	3.645	0.829		−0.184	0.088		−0.893	0.175
Org.F	780	1	5	3.716	0.990		−0.574	0.088		−0.806	0.175
Env.F	780	1	5	3.720	0.747		−0.487	0.088		−0.157	0.175

Env.F: Environmental Factors; Int.F: Interpersonal Factors; Ind.F: Individual Factors; Org.F: Organizational Factors; SP: Sports Participation.

#### 3.1.1 Reliability analysis and validity analysis

The reliability and validity of all measures were assessed. Cronbach’s alpha coefficients demonstrated high internal consistency: SP (α = 0.766), Ind.F (α = 0.971), Int.F (α = 0.975), Org.F (α = 0.939), and Env.F (α = 0.930), with an overall questionnaire reliability of 0.980. All values exceeded the acceptable threshold of 0.7, indicating satisfactory reliability. For validity assessment, the KMO measure of sampling adequacy was satisfactory for the overall scale (0.978) and SP (0.682), Ind.F (0.981), Int.F (0.980), Org.F (0.958), and Env.F (0.941). Additionally, Bartlett’s test of sphericity was significant (*p* < 0.001), confirming the data’s suitability for factor analysis (see Table 4).

**TABLE 4:** Reliability analysis and validity analysis.

Variables	*N* of items	*N*		Cronbach α
**Reliability statistics**				
SP	3	780		0.766
Ind.F	33	780		0.971
Int.F	33	780		0.975
Org.F	9	780		0.939
Env.F	19	780		0.930
Questionnaire as a whole	97	780		0.980
**Variables**	**KMO**	**Bartlett’s test of sphericity**		
		**Approx.chi-square**	**df**	**Sig.**
**KMO and bartlett test of variables**				
Questionnaire as a whole	0.978	67073.410	4656	0.000
SP	0.682	636.893	3	0.000
Ind.F	0.981	23914.358	528	0.000
Int.F	0.980	24603.657	528	0.000
Org.F	0.958	4898.141	36	0.000
Env.F	0.941	9714.983	171	0.000

Env.F: Environmental Factors; Int.F: Interpersonal Factors; Ind.F: Individual Factors; Org.F: Organizational Factors; SP: Sports Participation.

#### 3.1.2 Gender and grade differences

ANOVA revealed no significant gender differences in SP (*p* = 0.725), Int.F (*p* = 0.280), Org.F (*p* = 0.442), or Env.F (*p* = 0.424). However, significant gender differences were observed in Ind.F (*p* = 0.033), with males scoring higher (*M* = 3.91) than females (*M* = 3.79). Regarding grade differences, no significant variations were found in SP (*p* = 0.288), Ind.F (*p* = 0.219), or Org.F (*p* = 0.091). Significant grade-level differences emerged for Int.F (*F* = 3.309, *p* = 0.037) and Env.F (*F* = 4.383, *p* = 0.013). Both variables showed a consistent pattern of decreasing scores across advancing grades: for Int.F, 7th grade (*M* = 3.74) >8th grade (*M* = 3.63) >9th grade (*M* = 3.55); similarly for Env.F, 7th grade (*M* = 3.82) >8th grade (*M* = 3.71) >9th grade (*M* = 3.63) (see Table 5).

**TABLE 5:** Gender and grade differences.

	Gender (Mean ± Std. deviation)		*t*	*p*	
	Male (*n* = 404)	Female (*n* = 376)			
**Independent t-test**					
SP	4.90 ± 1.72	4.85 ± 1.70	0.352	0.725	
Ind.F	3.91 ± 0.80	3.79 ± 0.84	2.134	0.033*	
Int.F	3.61 ± 0.84	3.68 ± 0.82	−1.081	0.280	
Org.F	3.69 ± 1.02	3.74 ± 0.96	−0.769	0.442	
Env.F	3.74 ± 0.72	3.70 ± 0.77	0.8	0.424	
	**Grade (Mean ± Std. deviation)**			***F***	***p***
	**Junior high school grade one (*n* = 268)**	**Junior high school grade two (*n* = 272)**	**Junior high school grade three (*n* = 240)**		
**ANOVA**					
SP	4.99 ± 1.76	4.76 ± 1.74	4.89 ± 1.61	1.246	0.288
Ind.F	3.90 ± 0.79	3.87 ± 0.79	3.78 ± 0.89	1.523	0.219
Int.F	3.74 ± 0.86	3.63 ± 0.78	3.55 ± 0.85	3.309	0.037*
Org.F	3.82 ± 0.98	3.68 ± 0.98	3.64 ± 1.01	2.403	0.091
Env.F	3.82 ± 0.71	3.71 ± 0.77	3.63 ± 0.75	4.383	0.013*

Env.F: Environmental Factors; Int.F: Interpersonal Factors; Ind.F: Individual Factors; Org.F: Organizational Factors; SP: Sports Participation.

*p < 0.05, **p < 0.01.

### 3.2 Measurement model assessment

#### 3.2.1 Confirmatory factor analysis

Confirmatory factor analysis validated all measurement models. SP formed a saturated model (3 items). All other constructs demonstrated acceptable fit indices: CMIN/DF ranged from 2.785 to 4.839 (threshold: <5.0), GFI from 0.815 to 0.974 (threshold: >0.80), CFI from 0.922 to 0.987 (threshold: >0.90), and RMSEA from 0.048 to 0.070 (threshold: <0.08). Env.F showed the best fit (meeting more stringent thresholds of CMIN/DF <3.0 and RMSEA <0.05), while Int.F showed the lowest but still acceptable fit. All measurement models were deemed suitable for subsequent structural analysis (see Table 6).

**TABLE 6:** Model fit.

Index		CMIN/DF	GFI	IFI	TLI	CFI	RMSEA
Common index	Ideal	<3	>0.9	>0.9	>0.9	>0.9	<0.05
	Acceptable	<5	>0.8	>0.8	>0.8	>0.8	<0.08
**SP**							
Ind.F		3.624	0.856	0.946	0.942	0.945	0.058
Int.F		4.839	0.815	0.923	0.917	0.922	0.07
Org.F		3.358	0.974	0.987	0.983	0.987	0.055
Env.F		2.785	0.946	0.973	0.968	0.973	0.048

Env.F: Environmental Factors; Int.F: Interpersonal Factors; Ind.F: Individual Factors; Org.F: Organizational Factors; SP: Sports Participation.

#### 3.2.2 Convergent validity

Convergent validity was assessed using AVE and CR values. All constructs demonstrated good convergent validity with AVE values exceeding the recommended threshold of 0.5 and CR values above 0.7. Specifically, SP showed adequate convergent validity (AVE = 0.54, CR = 0.78). Ind.F, Int.F, Org.F and Env.F all demonstrated strong convergent validity with AVE values ranging from 0.59 to 0.73 and CR values ranging from 0.91 to 0.97. These results confirm that indicators within each construct adequately converge on their respective latent variables, supporting the measurement model’s validity (see Table 7).

**TABLE 7:** Convergent validity.

Path	Estimate	S.E.	C.R.	P	Std.Estimate	AVE	CR
**Convergence validity of confirmatory factor analysis**							
Y1 ← SP	1				0.809	0.538	0.775
Y2 ← SP	0.863	0.056	15.358	***	0.759		
Y3 ← SP	0.823	0.057	14.479	***	0.619		
X1 ← SAC	1				0.733	0.619	0.965
X2 ← SAC	0.904	0.048	18.786	***	0.664		
X3 ← SAC	1.087	0.048	22.642	***	0.79		
X4 ← SAC	1.08	0.048	22.592	***	0.789		
X5 ← SAC	1.115	0.048	23.171	***	0.807		
X6 ← SAC	1.12	0.049	23.013	***	0.802		
X7 ← SAC	1.143	0.049	23.111	***	0.805		
X8 ← SAC	1.145	0.049	23.541	***	0.819		
X9 ← SAC	1.113	0.048	23.072	***	0.804		
X10 ← SAC	1.063	0.048	22.151	***	0.774		
X11 ← SAC	1.141	0.047	24.064	***	0.835		
X12 ← SAC	1.138	0.048	23.727	***	0.825		
X13 ← SAC	1.109	0.05	22.246	***	0.777		
X14 ← SAC	1.084	0.049	22.09	***	0.772		
X15 ← SAC	1.071	0.049	22.027	***	0.77		
X16 ← SAC	1.097	0.049	22.302	***	0.779		
X17 ← SAC	1.16	0.05	23.364	***	0.813		
X18 ← PNOE	1				0.866	0.676	0.971
X19 ← PNOE	1.037	0.03	34.688	***	0.878		
X20 ← PNOE	0.978	0.032	30.683	***	0.824		
X21 ← PNOE	1.033	0.032	32.16	***	0.845		
X22 ← PNOE	0.9	0.033	26.9	***	0.764		
X23 ← PNOE	1.089	0.029	37.183	***	0.908		
X24 ← PNOE	0.788	0.035	22.679	***	0.683		
X25 ← PNOE	0.997	0.031	32.214	***	0.846		
X26 ← PNOE	0.983	0.031	32.154	***	0.845		
X27 ← PNOE	0.976	0.032	30.521	***	0.822		
X28 ← PNOE	1	0.031	32.341	***	0.848		
X29 ← PNOE	1.019	0.031	32.398	***	0.849		
X30 ← PNOE	0.762	0.033	23.054	***	0.691		
X31 ← PNOE	1.055	0.029	36.589	***	0.901		
X32 ← PNOE	1.019	0.03	34.074	***	0.871		
X33 ← PNOE	0.731	0.034	21.648	***	0.661		
F1 ← PS	1				0.757	0.622	0.97
F2 ← PS	1.105	0.047	23.7	***	0.791		
F3 ← PS	1.093	0.046	23.537	***	0.786		
F4 ← PS	1.043	0.045	23.411	***	0.782		
F5 ← PS	1.089	0.046	23.507	***	0.785		
F6 ← PS	1.171	0.047	25.012	***	0.827		
F7 ← PS	0.821	0.046	17.826	***	0.616		
F8 ← PS	0.884	0.045	19.519	***	0.668		
F9 ← PS	1.096	0.048	22.94	***	0.769		
F10 ← PS	1.099	0.048	23.031	***	0.772		
F11 ← PS	1.213	0.048	25.466	***	0.839		
F12 ← PS	1.183	0.048	24.651	***	0.817		
F13 ← PS	1.223	0.048	25.227	***	0.833		
F14 ← PS	1.206	0.048	25.009	***	0.827		
F15 ← PS	1.193	0.048	25.102	***	0.829		
F16 ← PS	1.148	0.047	24.553	***	0.814		
F17 ← PS	1.139	0.047	24.423	***	0.811		
F18 ← PS	1.17	0.047	24.731	***	0.819	0.693	0.967
F19 ← PS	1.126	0.047	24	***	0.799		
F20 ← PS	1.132	0.047	23.853	***	0.795		
F21 ← FS	1				0.864		
F22 ← FS	1.031	0.029	35.096	***	0.888		
F23 ← FS	0.944	0.029	32.274	***	0.851		
F24 ← FS	1.05	0.03	35.382	***	0.891		
F25 ← FS	0.879	0.029	30.015	***	0.818		
F26 ← FS	0.973	0.03	32.397	***	0.852		
F27 ← FS	0.789	0.03	26.434	***	0.759		
F28 ← FS	0.81	0.03	26.954	***	0.768		
F29 ← FS	0.817	0.03	27.321	***	0.774		
F30 ← FS	0.876	0.029	30.075	***	0.819		
F31 ← FS	0.891	0.028	31.279	***	0.837		
F32 ← FS	0.893	0.029	31.072	***	0.834		
F33 ← FS	0.961	0.03	32.472	***	0.853		
Z1 ← Org.F	1				0.794	0.63	0.939
Z2 ← Org.F	0.965	0.041	23.621	***	0.765		
Z3 ← Org.F	1.018	0.04	25.166	***	0.803		
Z4 ← Org.F	1.096	0.042	26.284	***	0.829		
Z5 ← Org.F	1.1	0.041	26.524	***	0.835		
Z6 ← Org.F	1.057	0.042	25.073	***	0.801		
Z7 ← Org.F	1.017	0.042	24.068	***	0.776		
Z8 ← Org.F	0.99	0.041	24.373	***	0.784		
Z9 ← Org.F	0.993	0.043	23.212	***	0.755		
H1 ← FEI	1				0.754	0.589	0.909
H2 ← FEI	0.993	0.052	18.955	***	0.672		
H3 ← FEI	0.888	0.047	18.729	***	0.665		
H4 ← FEI	1.1	0.047	23.544	***	0.815		
H5 ← FEI	1.299	0.053	24.734	***	0.851		
H6 ← FEI	1.102	0.052	21.063	***	0.739		
H7 ← FEI	1.373	0.055	24.878	***	0.855		
H8 ← CPE	1				0.696	0.586	0.849
H9 ← CPE	1.051	0.057	18.31	***	0.733		
H10 ← CPE	1.221	0.06	20.286	***	0.832		
H11 ← CPE	1.207	0.062	19.566	***	0.793		
H12 ← CRE	1				0.778	0.726	0.914
H13 ← CRE	1.157	0.045	25.48	***	0.837		
H14 ← CRE	1.261	0.045	28.114	***	0.908		
H15 ← CRE	1.283	0.047	27.093	***	0.879		
H16 ← CSSE	1				0.819	0.684	0.896
H17 ← CSSE	1.004	0.035	28.511	***	0.871		
H18 ← CSSE	1.063	0.038	28.067	***	0.861		
H19 ← CSSE	0.879	0.038	23.292	***	0.751		

Note: CPE, Community Public Service Facilities; CRE, Aesthetic Evaluation of Residential Environment; CSSE, Community Sports Service Facilities; FEI, Family Exercise Equipment Ownership and Utilization; FS, Friend Support for Sports Participation; Org.F, Organizational Factors; PNOE, Perceived Positive and Negative Outcome Expectations; PS, Parental Support for Sports Participation; SAC, Self-assessment of Confidence in Sports Participation; SP, Sports Participation.

#### 3.2.3 Discriminant validity

Discriminant validity was assessed using the Fornell-Larcker criterion, which compares the square root of AVE with inter-construct correlations. For Ind.F, the square root of AVE for SAC (0.787) and PONE (0.822) exceeded their correlation (0.564), confirming discriminant validity. Similarly, for Int.F, the square root of AVE for PS (0.789) and FS (0.832) was greater than their correlation (0.664). For Env.F, all diagonal values (square roots of AVE) were larger than corresponding off-diagonal correlation coefficients: FEI (0.768), CPE (0.765), CRE (0.852), and CSSE (0.827). Correlation coefficients between Env.F dimensions ranged from 0.395 to 0.626, all significant at *p* < 0.01 level. These results demonstrate that each construct captured unique variance not explained by other constructs in the model, establishing discriminant validity among all factors (see Table 8).

**TABLE 8:** Discriminant validity.

Discriminant validity: Pearson correlation and square root of AVE				
**Ind.F**				
	**SAC**	**PNOE**		
SAC	**0.787**			
PNOE	0.564**	**0.822**		
**Int.F**				
	**PS**	**FS**		
PS	**0.789**			
FS	0.664**	**0.832**		
**Env.F**				
	**FEI**	**CPE**	**CRE**	**CSSE**
FEI	**0.768**	**0.765**	**0.852**	**0.827**
CPE	0.444**			
CRE	0.395**	0.578**		
CSSE	0.626**	0.497**	0.497**	

Note: SAC, Self-assessment of Confidence in Sports Participation; PNOE, Positive and Negative Outcome Expectations in Sports Participation; PS, Parental Support for Sports Participation; FS, Friend Support for Sports Participation; FEI, Family Exercise Equipment Ownership and Utilization; CPE, Community Public Service Facilities; CRE, Aesthetic Evaluation of Residential Environment; CSSE, Community Sports Service Facilities. Diagonal figures are the square root values of ave.

*p < 0.05, **p < 0.01.

#### 3.2.4 Correlation analysis

**FIGURE 2:**
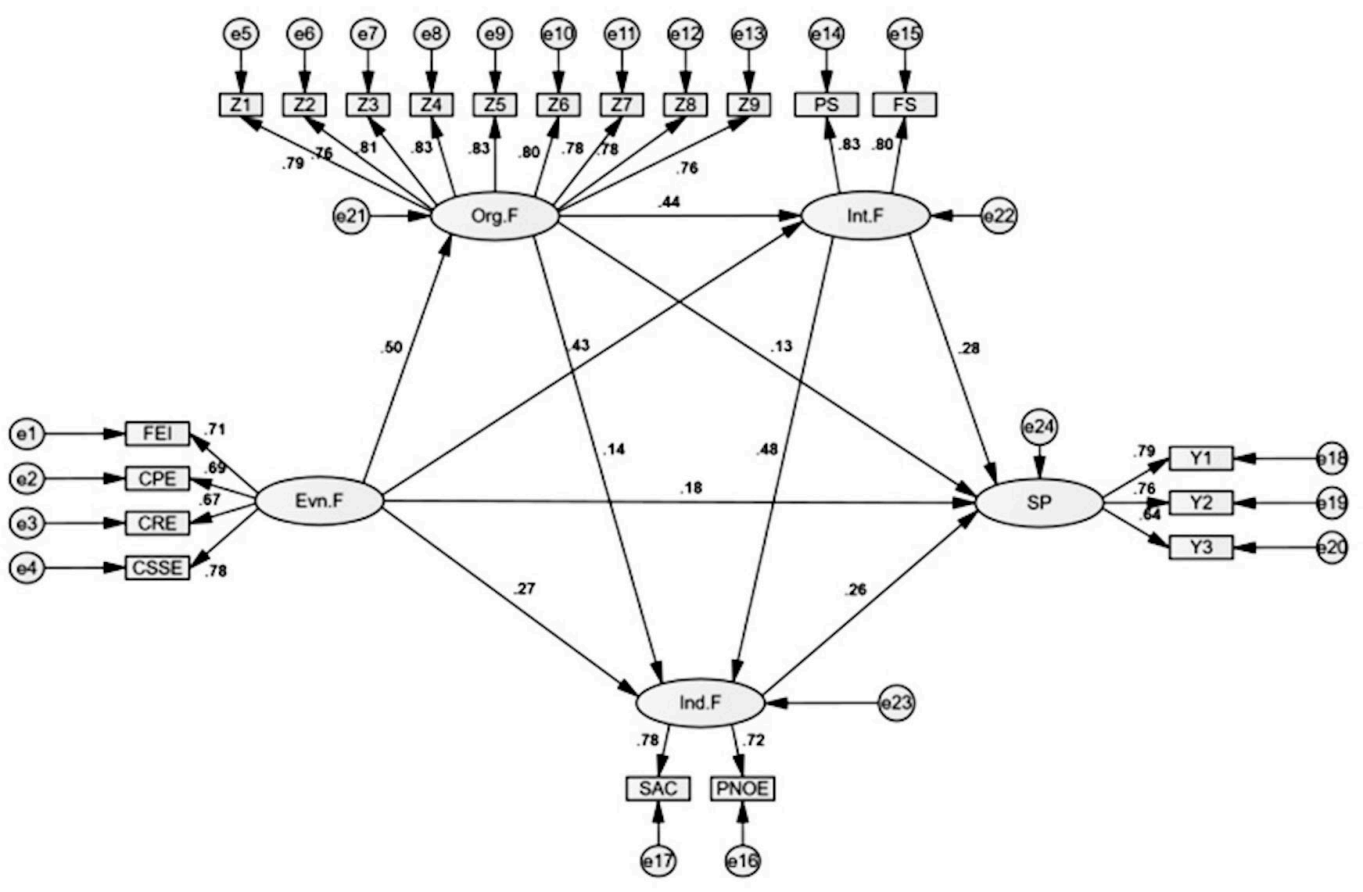
Structural equation modeling of factors influencing sports participation in adolescents. Note: CPE, Community Public Service Facilities; CRE, Aesthetic Evaluation of Residential Environment; CSSE, Community Sports Service Facilities; Env.F, Environmental Factors; FEI, Family Exercise Equipment Ownership and Utilization; FS, Friend Support for Sports Participation; Ind.F, Individual Factors; Int.F, Interpersonal Factors; Org.F, Organizational Factors; PS, Parental Support for Sports Participation; PNOE, Positive and Negative Outcome Expectations in Sports Participation; SAC, Self-assessment of Confidence in Sports Participation; SP, Sports Participation.

Pearson correlation analysis was conducted to examine relationships among all constructs. Results revealed significant positive correlations between all variables (*p* < 0.01). SP was positively correlated with Ind.F (*r* = 0.491), Int.F (*r* = 0.531), Org.F (*r* = 0.476), and Env.F (*r* = 0.488). Ind.F showed significant positive correlations with Int.F (*r* = 0.567), Org.F (*r* = 0.484), and Env.F (*r* = 0.493). Int.F demonstrated significant positive associations with Org.F (*r* = 0.563) and Env.F (*r* = 0.515). Finally, Org.F and Env.F were positively correlated (*r* = 0.415). These significant correlations provide preliminary support for the hypothesized relationships in the structural model while remaining below thresholds that would indicate multicollinearity concerns. Based on these findings, a structural equation model was developed to further examine the proposed relationships among variables (see Table 9). The final structural model with standardized path coefficients is shown in Figure 2.

**TABLE 9:** Correlation analysis.

	SP	Ind.F	Int.F	Org.F	Env.F
**Pearson correlation**					
SP	1	1	1	1	1
Ind.F	0.491**				
Int.F	0.531**	0.567**			
Org.F	0.476**	0.484**	0.563**		
Env.F	0.488**	0.493**	0.515**	0.415**	

Note: Env.F, Environmental Factors; Int.F, Interpersonal Factors; Ind.F, Individual Factors; Org.F, Organizational Factors; SP, Sports Participation.

* *p* < 0.05, ** *p* < 0.01.

### 3.3 Structural equation modeling

Figure 1 illustrates the structural model. Different measurement approaches were employed based on indicator complexity. For Org.F, all original items were retained as indicators. For the remaining factors with numerous items, parceling was implemented: Ind.F was parceled into self-assessment of confidence (SAC) and positive/negative outcome expectations (PONE); Int.F was parceled into parental support (PS) and peer support (FS); and Env.F was parceled into family equipment inventory (FEI), community public facilities (CPE), community residential environment (CRE), and community sports service facilities (CSSE).

#### 3.3.1 Model fit

The structural model demonstrated excellent fit to the data. The normed chi-square (CMIN/DF = 2.601) was below the threshold of 3.0, indicating acceptable fit. All comparative fit indices exceeded recommended thresholds for good fit: GFI = 0.949, IFI = 0.971, TLI = 0.965, and CFI = 0.971 (all >0.95). The RMSEA value of 0.045 was below 0.05, meeting the criterion for good fit. These indices collectively confirmed that the hypothesized structural model adequately represented the empirical data, supporting further analysis of the structural relationships (see Table 10).

**TABLE 10:** Structural model fit indices.

Index		CMIN/DF	GFI	IFI	TLI	CFI	RMSEA
Common index	Ideal	<3	>0.9	>0.9	>0.9	>0.9	<0.05
	Acceptable	<5	>0.8	>0.8	>0.8	>0.8	<0.08
Measurement model fitting result		2.601	0.949	0.971	0.965	0.971	0.045

#### 3.3.2 Path analysis

The path analysis revealed significant direct effects across all hypothesized relationships in the structural model. Env.F demonstrated significant positive effects on Org.F, (β = 0.502, *p* < 0.001), Int.F (β = 0.428, *p* < 0.001), Ind.F (Ind.F, β = 0.267, *p* < 0.001), and Sports Participation (SP, β = 0.179, *p* = 0.002).

Org.F exhibited significant positive influences on Int.F (β = 0.436, *p* < 0.001), Ind.F (β = 0.140, *p* = 0.005), and SP (β = 0.130, *p* = 0.007). Interpersonal Factors positively affected both Ind.F (β = 0.481, *p* < 0.001) and SP (β = 0.280, *p* < 0.001). Finally, Ind.F significantly influenced Sports Participation (β = 0.258, *p* < 0.001).

These results indicate that environmental, organizational, interpersonal, and Ind.F all contribute significantly to adolescents’ sports participation, with both direct and indirect pathways of influence operating through the hypothesized model (see Table 11).

**TABLE 11:** Path test.

Path	Estimate	S.E.	C.R.	*P*	STD.Estimate
Org.F ← Env.F	0.632	0.055	11.492	***	0.502
Int.F ← Env.F	0.394	0.042	9.444	***	0.428
Int.F ← Org.F	0.319	0.030	10.520	***	0.436
Ind.F ← Env.F	0.247	0.052	4.765	***	0.267
Ind.F ← Org.F	0.103	0.037	2.809	0.005	0.140
Ind.F ← Int.F	0.483	0.070	6.925	***	0.481
SP ← Env.F	0.395	0.125	3.158	0.002	0.179
SP ← Org.F	0.228	0.084	2.705	0.007	0.130
SP ← Int.F	0.672	0.185	3.632	***	0.280
SP ← Ind.F	0.615	0.184	3.340	***	0.258

Note: Env.F, Environmental Factors; Int.F, Interpersonal Factors; Ind.F, Individual Factors; Org.F, Organizational Factors; SP, Sports Participation.

### 3.4 Mediation analysis

Bootstrapped indirect effects and bias-corrected 95% confidence intervals are presented in Table 12.

**TABLE 12:** A test of the mediating role of Org.F, Int.F and Ind.F in the influence of Env.F on SP.

Effect type	Path	Effects	BootSE			Bias-corrected 95%CI			
						BootLLCI			BootULCI
Total effect	Env.F → SP	0.592	0.035			0.522			0.659
Direct effect	Env.F → SP	0.179	0.060			0.057			0.300
Indirect effect	Env.F → Org.F → SP	0.065	0.028			0.011			0.120
	Env.F → Int.F → SP	0.120	0.041			0.046			0.207
	Env.F → Ind.F → SP	0.069	0.028			0.023			0.134
	Env.F → Org.F → Int.F → SP	0.061	0.021			0.024			0.109
	Env.F → Org.F → Ind.F → SP	0.018	0.010			0.004			0.044
	Env.F → Int.F → Ind.F → SP	0.053	0.020			0.020			0.100
	Env.F → Org.F → Int.F → Ind.F → SP	0.027	0.010			0.010			0.052
**A test of the mediating role of Int.F and Ind.F in the influence of Org.F on SP.**									
Total effect	Org.F → SP	0.342	0.043		0.257			0.425	
Direct effect	Org.F → SP	0.130	0.055		0.020			0.233	
Indirect effect	Org.F → Int.F → SP	0.122	0.040		0.048			0.209	
	Org.F → Ind.F → SP	0.036	0.020		0.007			0.086	
	Org.F → Int.F → Ind.F → SP	0.054	0.020		0.020			0.101	
**A test of the mediating role of Ind.F in the influence of Int.F on SP.**									
Total effect	Int.F → SP	0.404	0.070	0.264			0.538		
Direct effect	Int.F → SP	0.280	0.088	0.108			0.456		
	Int.F → Ind.F → SP	0.124	0.045	0.043			0.221		

Note: Env.F, Environmental Factors; Int.F, Interpersonal Factors; Ind.F, Individual Factors; Org.F, Organizational Factors; SP, Sports Participation.

#### 3.4.1 Environmental factors to sports participation

Multiple mediation pathways from Env.F to SP were examined. Simple mediation effects were confirmed through Org.F [β = 0.065, 95% CI (0.011, 0.120)], Int.F [β = 0.120, 95% CI (0.046, 0.207)], and Ind.F [β = 0.069, 95% CI (0.023, 0.134)]. With the direct effect remaining significant [β = 0.179, 95% CI (0.057, 0.300)], these results indicate partial mediation through all three factors.

Additionally, significant serial mediation effects were found through: Org.F → Int.F [β = 0.061, 95% CI (0.024, 0.109)], Org.F → Ind.F [β = 0.018, 95% CI (0.004, 0.044)], Int.F → Ind.F [β = 0.053, 95% CI (0.020, 0.100)], and the three-path mediation Org.F → Int.F → Ind.F [β = 0.027, 95% CI (0.010, 0.052)].

#### 3.4.2 Organizational factors to sports participation

Org.F’s influence on SP was partially mediated through Int.F [β = 0.122, 95% CI (0.048, 0.209)] and Ind.F [β = 0.036, 95% CI (0.007, 0.086)], with the direct effect remaining significant [β = 0.130, 95% CI (0.020, 0.233)]. Serial mediation through Int.F → Ind.F was also significant [β = 0.054, 95% CI (0.020, 0.101)].

#### 3.4.3 Interpersonal factors to sports participation

Int.F’s effect on SP was partially mediated by Ind.F [β = 0.124, 95% CI (0.043, 0.221)], with the direct effect remaining significant [β = 0.280, 95% CI (0.108, 0.456)].

## 4 Discussion

This study aimed to examine adolescents’ SP based on Bronfenbrenner’s ecological health theory through a four-level SEM. We hypothesized that sports participation is not determined by a single factor but results from the dynamic interaction of environmental, organizational, interpersonal, and individual domains. The results support this hypothesis: all four levels were significantly associated with SP (*r* = 0.476–0.531, *p* < 0.01). SEM revealed both direct and indirect pathways, and the overall model fit indices were within acceptable ranges (CMIN/DF = 2.601, GFI = 0.949, IFI = 0.971, TLI = 0.965, CFI = 0.971, RMSEA = 0.045). Further analysis indicated that the environment influenced SP by activating organizational resources, and organizations shaped interpersonal relationships and enhanced individual psychological efficacy. Path analysis showed that all path coefficients were statistically significant, highlighting the key mediating role of individual psychological resources, such as self-efficacy (the magnitude of these β coefficients indicates the relative strength of each influence, which can guide intervention priorities: the higher β for interpersonal factors suggests that family and peer support may have the most immediate impact on adolescents’ SP).

Notably, the results indicated gender and grade differences. Ind.F differed significantly by gender (*p* = 0.033), consistent with traditional socialization patterns: males exhibited stronger athletic identity (mean *t* = 3.91), whereas females were implicitly constrained by the “feminine ideal” (Messner, 2002). Academic pressure across different grades significantly affected interpersonal and environmental factors (*p* < 0.05), with support networks gradually weakening as academic demands increased (e.g., interpersonal support Grade 1 *F* = 3.74 vs. Grade 2 *F* = 3.63; environmental perception *F* = 3.63). Interestingly, individual psychological resilience showed only a slight, non-significant decline (*F* = 1.523, *p* = 0.219), suggesting that adolescents could partially buffer external resource weakening through internal regulation. These differences indicate that sports participation cannot be understood outside socio-cultural and educational contexts and underscore the importance of individual psychological mechanisms in maintaining sustained engagement.

### 4.1 Environmental factors

Among the four levels, Env.F had the broadest influence. SEM results showed that the environment not only directly predicted SP (β = 0.179, *p* < 0.01) but also significantly shaped organizational (β = 0.502), interpersonal (β = 0.428), and individual (β = 0.267) factors. Bootstrap mediation tests further revealed significant chain effects (confidence intervals did not include zero). Thus, although the direct path coefficient of the environment was not the strongest, it forms the foundation for other paths. (This means that environmental improvements alone may produce modest immediate effects on SP, but by facilitating schools and social networks, the indirect impact can be substantial, highlighting the strategic value of community infrastructure and resource investment.) National fitness policies play a key role in promoting community health (Dong et al., 2025). The Healthy China 2030 plan (Central Committee of the Communist Party of China and State Council of the People’s Republic of China, 2016) further emphasizes community-based fitness promotion as a means of achieving universal health goals.

The practical implication is that when environmental infrastructure and public sports investment are prioritized, schools, peers, and families can more easily work together to promote adolescent sports participation. International experience shows that urban planning secures extracurricular exercise by maintaining greenways, parks, and subsidizing sports clubs (Salvo et al., 2021; Smith et al., 2017). Conversely, compared with large cities, the construction and operation of public sports facilities in some small- and medium-sized cities and rural areas still face challenges, which may limit opportunities for adolescents’ extracurricular sports activities. This imbalance highlights the necessity of environmental interventions as a foundation for public health strategies.

### 4.2 Organizational factors

Org.F (e.g., school policies, curricula, facilities, teacher capabilities) directly predicted SP (β = 0.130, *p* < 0.01) and indirectly influenced SP through interpersonal and individual mediators (Env.F → Org.F → Int.F/Ind.F → SP). Although the direct β is smaller than for other factors, the organizational pathways amplify environmental inputs, meaning that school-level interventions can magnify broader environmental improvements. Thus, schools serve as an important organizational link between the environment and individual behavior (Tan, 2024). Empirical evidence demonstrates that by investing in teacher training, facility development, and extracurricular programs, schools can promote adolescents’ athletic skill development and strengthen their sense of group belonging (Grigoroiu et al., 2024; Sulz et al., 2023).

However, there is a gap in policy implementation. Despite the “Double Reduction” policy State Council of the PRC (State Council of the People’s Republic of China, 2021) and the “Sports Power Nation Strategy” (Wang and Zheng, 2022) advocating guaranteed physical education hours and enriched extracurricular activities, many schools still weaken sports education due to exam pressure or insufficient resources (Feraco et al., 2023; Singla et al., 2021). In contrast, Nordic and North American systems institutionalize sports through assessment systems and club structures to ensure long-term sustainability (Government of Canada, 2025). Our study quantitatively supports this: without organizational support, both interpersonal support and individual efficacy weaken, reducing adolescents’ ability to maintain ongoing sports participation (the β values suggest that even modest improvements at the organizational level can indirectly enhance SP by strengthening Int.F and Ind.F).

### 4.3 Interpersonal factors

Int.F had the strongest effect on Ind.F (β = 0.481, *p* < 0.001) and directly promoted SP (β = 0.280, *p* < 0.001). The relatively high β indicates that interventions targeting family and peer support may yield the largest immediate gains in adolescents’ self-efficacy and sports engagement. This confirms that parental involvement, peer encouragement, and teacher-student relationships are the most direct motivational sources for adolescent sports participation (van der Sluis et al., 2025).

In middle school, peer belonging and parental emphasis on sports are particularly critical. Interpersonal factors also mediate between organizational and Ind.F, explaining how institutional and environmental resources are “translated” into adolescents’ daily experiences.

Cultural context should not be overlooked. In China, parental and peer support often centers on academics, relegating sports to a secondary status (Liu and Zhan, 2020). In contrast, Japan’s “club activities” and the Western “school teams + community clubs” models provide adolescents with long-term sports social networks, fostering emotional belonging and team benefits (Liu and Zhan, 2020). Our mediation results similarly indicate that interpersonal support not only strengthens immediate participation but also converts into long-term behavior by enhancing self-confidence and resilience (Shi et al., 2025). Therefore, transforming parents’ and peers’ sports perceptions to equal importance with academics is key to sustaining adolescents’ sports motivation.

### 4.4 Individual factors

Ind.F are the “final link” in the chain. Self-efficacy and positive expectations significantly predicted SP (β = 0.258, *p* < 0.001) and mediated the pathways from environment, organization, and interpersonal factors to SP. (The β value highlights that increasing adolescents’ self-efficacy may produce substantial and lasting improvements in sports participation, reinforcing the importance of cognitive and psychological interventions.) This confirms that adolescents’ sports behavior relies on the belief “I can do it” and the support of psychological resilience (Hulteen et al., 2018; Tammelin et al., 2003).

Cross-national comparisons highlight challenges in China. European countries cultivate long-term adherence through physical fitness assessments and early sports initiation programs (Ortega et al., 2022), whereas Chinese adolescents often face fragmented or exam-driven opportunities for exercise (Cai et al., 2025). Therefore, when encountering injury or academic conflicts, they struggle to maintain self-efficacy without systematic support (Shi et al., 2025). This study suggests that enhancing individual resilience requires not only motivational training but also cognitive interventions that reframe sports as a long-term health investment rather than a temporary or exam-oriented task (Tao, 2023).

### 4.5 Implications of the research

Overall, adolescent sports participation results from the interplay of environmental, organizational, interpersonal, and individual factors. Environmental factors serve as the foundation, activating schools and community organizations through resources and facilities. Organizational factors further shape interpersonal relationships and individual psychological efficacy, while the sustainability of sports participation ultimately depends on individual self-efficacy and positive expectations. Policy implications should act on both macro and micro levels. At the community level, investment in sports infrastructure should be increased to improve accessibility, safety, and diversity, encouraging adolescents from different regions to engage in sports and expand opportunities. At the school level, physical education should be institutionalized through curriculum design, assessment systems, and teacher support to provide an environment and support for continuous participation. At the family and peer level, cultural perceptions should be transformed to view sports as equally important as academics, enhancing adolescents’ motivation and positive expectations through parental modeling and peer support. The relative β values suggest that interventions focusing on interpersonal and individual factors may yield quicker and more substantial effects, while environmental and organizational improvements are essential for sustained and systemic changes. In resource-limited contexts, broad environmental interventions may face constraints such as insufficient funding, limited access to facilities, or uneven public space distribution. Accordingly, we recommend prioritize key organizational resources and gradually enhance environmental support as resources permit. Feasible strategies include phased facility development, partnerships with local organizations, use of school grounds for after-hours activities, and promotion of low-cost or home-based physical activities, which can optimize limited resources while ensuring equitable opportunities for adolescent sports participation. These integrated measures can strengthen individual psychological efficacy, enhance the continuity of sports behavior, and provide scientific evidence for public health interventions.

## 5 Conclusion

This study demonstrates that adolescents’ sports participation in China is shaped by the dynamic interplay of environmental, organizational, interpersonal, and individual factors, with interpersonal support and self-efficacy exerting the strongest direct effects, while environmental and organizational factors, although their direct effects are relatively small, remain statistically significant and also exert important indirect effects through interpersonal and individual pathways, thereby amplifying overall influence and forming a multi-level mechanism. Translating these insights into practice, we propose a coordinated, multilevel agenda that strengthens family–peer supports through school–family–community initiatives and low-barrier joint activities; enhances individual self-efficacy via graduated goal setting, structured feedback, and self-monitoring tools that emphasize self-referenced progress; institutionalizes school-level measures by embedding daily MVPA targets within curricula, diversifying clubs and electives, extending facility access beyond school hours, and coordinating teacher–coach–volunteer teams; improves community environments by expanding safe, affordable, and proximate facilities and staging visible campaigns to reinforce supportive norms; and aligns policy and cross-sector mechanisms by linking school accountability to physical activity indicators, incentivizing education–health–urban planning partnerships, funding shared-use agreements and evidence-based programs, and embedding continuous monitoring and evaluation (e.g., weekly MVPA, attendance, self-efficacy and perceived support). Collectively, these integrated strategies are expected to bolster adolescents’ competence and motivation, foster sustained engagement in physical activity, and provide actionable evidence to inform public health and educational policy.

## 6 Limitations

This study has several limitations that should be acknowledged. First, the cross-sectional design precludes causal inference, making it impossible to establish temporal ordering or rule out reverse causality; longitudinal or experimental designs are needed to capture changes in adolescents’ sport participation over time. Second, the sample was drawn exclusively from schools in Tianjin—a highly developed urban center—so generalizability to rural or less affluent regions is limited; moreover, socioeconomic covariates (e.g., parental education, household income, urban–rural status) were not included and may confound the observed associations. Third, all measures were self-administered and self-reported, which can introduce response and recall biases (e.g., over- or under-estimating participation and supports) and subjective judgment; future work should incorporate objective indicators (e.g., wearables, activity logs, direct observation) and triangulate multiple data sources to improve accuracy and reliability. Although we implemented procedural and statistical remedies for common-method bias, residual method variance cannot be fully ruled out. Fourth, the operationalization of environmental, organizational, interpersonal, and individual constructs was necessarily simplified; finer-grained dimensions (e.g., teacher–student relationship quality, peer network structure, social capital, community involvement) warrant examination. Finally, while gender and grade differences were observed, we did not conduct multi-group SEM, measurement invariance testing, or other robustness checks to probe moderation; addressing these issues will enhance the robustness, ecological validity, and practical relevance of multilevel models of adolescent sport participation.

## Data Availability

The raw data supporting the conclusions of this article will be made available by the corresponding author upon reasonable request. Requests to access the datasets should be directed to XG, 201290261@tjgydx.wecom.work.
